# A new species of the genus *Hylcalosia* Fischer (Hymenoptera: Braconidae: Alysiinae) from South Korea, with a key to the Korean species

**DOI:** 10.3897/zookeys.1070.73377

**Published:** 2021-11-10

**Authors:** Ju-Hyeong Sohn, Cornelis van Achterberg*, Yunjong Han, Hyojoong Kim

**Affiliations:** 1 Animal Systematics Lab., Department of Biology, Kunsan National University, Gunsan, 54150, Republic of Korea Kunsan National University Gunsan Republic of Korea; 2 State Key Laboratory of Rice Biology and Ministry of Agriculture / Key Lab of Agricultural Entomology, Institute of Insect Science, Zhejiang University, Hangzhou, 310058, China Zhejiang University Hangzhou China

**Keywords:** COI barcode, cyclostome, koinobiont, natural enemy, parasitoid wasp, systematics, taxonomy

## Abstract

The species of the genus *Hylcalosia* Fischer, 1967 (Braconidae: Alysiinae) from South Korea are revised. One species, *Hylcalosiabicolor***sp. nov.**, is new to science. They are described and illustrated herein and an identification key to the Korean species is added. In addition, the DNA barcode region of the mitochondrial cytochrome c oxidase subunit I (COI) has been analysed for the new species and *H.sutchanica* is used for genetic comparison.

## Introduction

The subfamily Alysiinae, which is one of the large taxa in the family Braconidae, occurs worldwide and contains over 2,440 valid species (Yu et al. 2016). In Korea, 180 species in 21 genera are listed in the National Species List of South Korea (NIBR 2019). This group can be discriminated from other subfamilies by having non-overlapping mandibles and is subdivided into two tribes, Alysiini and Dacnusini, which are distinguished from each other by the presence or absence of fore wing vein r-m, respectively (Shaw and Huddleston 1991). Alysiinae are known as koinobiont endoparasitoids of dipteran larvae, characteristically using their mandible (with three or four teeth, rarely more or less) to break open the puparium of the host.

*Hylcalosia* Fischer, 1967 is a small genus of Alysiinae, which includes 18 species (Yu et al. 2016, Yao et al. 2020). This genus is easily diagnosed by the rugose or granulated second and third metasomal tergites combined by the acutely protruding clypeus and enlarged upper valve of the ovipositor (van Achterberg 1983). Fischer (1967) re-described the type species from Myanmar *Holcalysiaruficeps* Cameron, 1910. Van Achterberg (1983) revised the genus *Hylcalosia* and described two new species: *H.maetoi* and *H.hemiflava* from Japan and Indonesia, respectively. Belokobylskij (1992) added two new species from Russia: *H.hymaenei* and *H.sutchanica*. Papp (1994) described *H.adsimilis* from North Korea and Chen & Wu (1994) *H.complexa* from China. Fischer (2008) added *H.laosensis* as a new species from Laos and Zheng et al. (2012)*H.ventisulcata* as a new species in China. Belokobylskij (2015) revised the Russian *Hylcalosia* species and synonymised *H.adsimilis* with *H.sutchanica*, reported *H.maetoi* from South Korea and described a new species, *H.livadiae*. Finally, four new species (*H.carinata*, *H.melasaraia*, *H.poricrenulata* and *H.verticalis*) were described from China by Zhu et al. (2018) and five new species (*H.bothynis*, *H.dichromata*, *H.eurykephale*, *H.leura* and *H.perkna*) from Thailand by Yao et al. (2020).

In this study, we present new morphological characters and the barcoding sequences of the COI region of *H.bicolor* sp. nov. and one previously-recorded species, *H.sutchanica*. Descriptions, diagnoses, an identification key and photographs of the diagnostic characters are provided.

## Materials and methods

Samples used in this study were collected with Malaise traps in South Korea at the DMZ Botanical Garden, Mandae-ri, Haean-myeon, Yanggu-gun, Gangwon-do. Sorting and preparation were done at the Animal Systematics Lab. (ASL), Department of Biology, Kunsan National University (KSNU) at Gunsan. For morphological identification, Zhu et al. (2017, 2018) and Yao et al. (2020) were used. Morphological characters were observed with a Leica M205C stereomicroscope. The Taxapad database (Yu et al. 2016) was used for references. We followed the terminology of Wharton (2002) and van Achterberg (1993). The type specimens are deposited in Korea National Arboretum (KNA).

A LEICA DMC2900 digital camera and a LEICA M205C stereomicroscope (Leica Geosystems AG) were used for photography and several pictures were taken for each height using multi-focusing technology. LAS V4.11 (Leica Geosystems AG, Wetzlar, Germany) and HeliconFocus 7 (Helicon Soft, Kharkiv, Ukraine) software were used for stacking work. After stacking work, illustrations were created using Adobe Photoshop CS6.

Extraction of DNA was done in ASL, KSNU. Whole genomic DNA was extracted from the specimens by using a DNeasy Blood & Tissue kit (QIAGEN Inc., Dusseldorf, Germany) following the manufacturer’s protocol. In order to conserve morphologically-complete voucher specimens, the DNA extraction method was used slightly modified from the ‘non-destructive method’ by Favret (2005) and ‘freezing method’ by Yaakop et al. (2009). In the original protocol, the sample was crushed or wounded and then soaked with 180 μl of buffer ATL + 20 μl of proteinase, followed by three hours incubation at 55°C. In slightly modified DNA extraction methods, samples were soaked with 180 μl of buffer ATL + 20 μl of proteinase K without destroying the sample, followed by 20 minutes incubation at 55°C and then kept in a freezer at -21°C overnight. After that, the general protocol was used for the remaining steps. The primer-set of LCO-1490 (5’-GGTCAACAAATCATAAAGATATTGG-3’) and HCO-2198 (5’-TAAACTTCAGGGTGACCAAAAAATCA-3’) was used to amplify approximately 658 bp as the partial front region of the COI. The polymerase chain reaction (PCR) products were amplified by using AccuPowerH PCR PreMix (BIONEER, Corp., Daejeon, Korea) in 20 μl reaction mixtures containing 0.4 μM of each primer, 20 μM of dNTPs, 20 μM of MgCl_2_ and 0.05 μg of the genomic DNA template. PCR amplification was performed using a GS1 thermo-cycler (Gene Technologies, Ltd., Essex, UK) according to the following procedure: initial denaturation at 95°C for 5 min, followed by 34 cycles at 94°C for 35 sec; an annealing temperature of 48°C for 25 sec; an extension at 72°C for 45 sec and a final extension at 72°C for 5 min. The PCR products were visualised by electrophoresis on a 1.5% agarose gel. A single band was observed, purified using a QIAquick PCR purification kit (QIAGEN, Inc., Milan, Italy) and then sequenced directly using an automated sequencer (ABI Prism 3730 XL DNA Analyzer) at Macrogen Inc. (Seoul, South Korea).

Sequence alignments were performed in MEGA version 7 (Kumar et al. 2016) with the ClustalW tool. To estimate the pairwise genetic distances, the *P*-distance model was conducted using MEGA version 7.

**Table 1.** COI pairwise genetic distances between two *Hylcalosia* species from South Korea.

	*Hylcalosiasutchanica*	*Hylcalosiabicolor*
***Hylcalosiasutchanica***	0.00	
***Hylcalosiabicolor***	0.091	0.00

## Results

A total of 563 bp of the COI barcode region were sequenced from *H.bicolor* sp. nov. and *H.sutchanica* which were deposited in GenBank (accession numbers MZ717196, MZ717194). Pairwise distances were estimated by using the *P*-distance model with the option for pairwise deletion. As a result, *H.bicolor* sp. nov. showed a fairly large genetic difference of 6% from *H.sutchanica*.

### Hylcalosia

Taxon classificationAnimaliaHymenopteraBraconidae

Fischer, 1967

2F148AFE-689D-5BEC-92AE-243BB475FF85

Holcalysia Cameron, 1910: 6 [nec Cameron, 1905]; Shenefelt, 1974: 993. Type species: Holcalysiaruficeps Cameron, 1910.
Hylcalosia Fischer, 1967: 125; Shenefelt, 1974: 993; Chen & Wu, 1994: 85; Belokobylskij, 1998: 297; Zheng, Chen & Yang, 2012: 454; Belokobylskij, 2015: 530. Type species: Holcalysiaruficeps Cameron, 1910.

#### Diagnosis.

**Figure 1.**
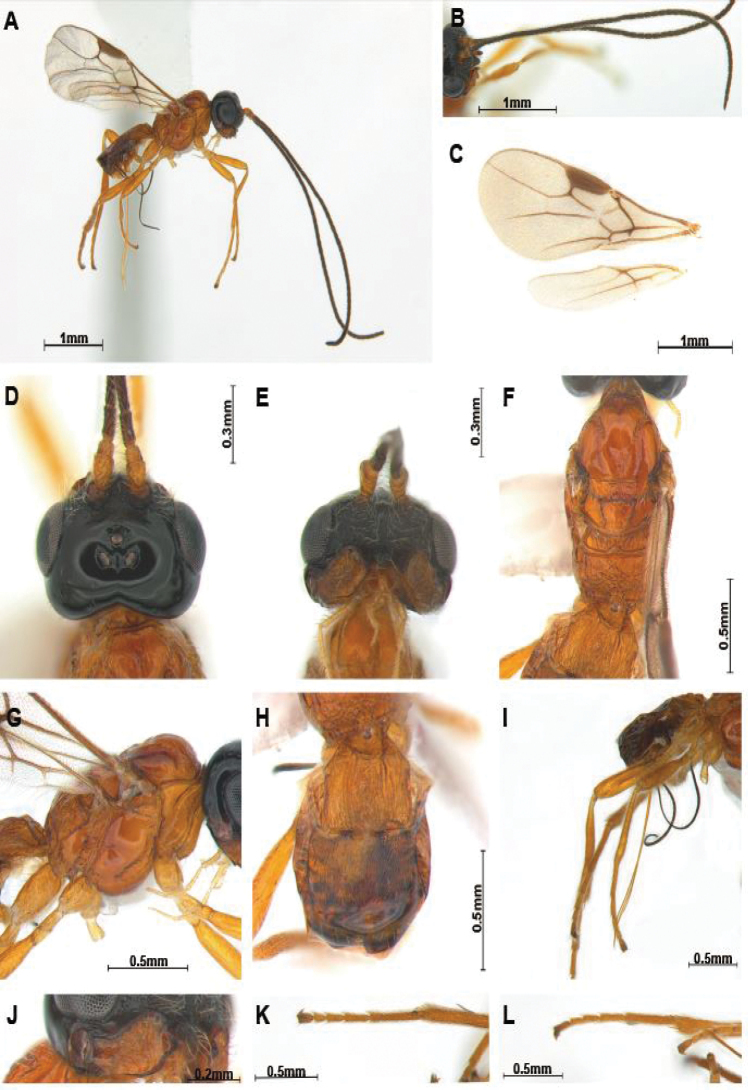
*Hylcalosiabicolor* sp. nov., ♀, holotype **A** body, lateral view **B** antennae **C** wings **D** head, dorsal view **E** head, ventral view **F** mesosoma, dorsal view **G** mesosoma, lateral view **H** propodeum and first metasomal tergite, dorsal view **I** ovipositor and hind leg, lateral view **J** mandible, lateral view **K** hind tarsus, dorsal view **L** hind tarsus, lateral view.

**Figure 2.**
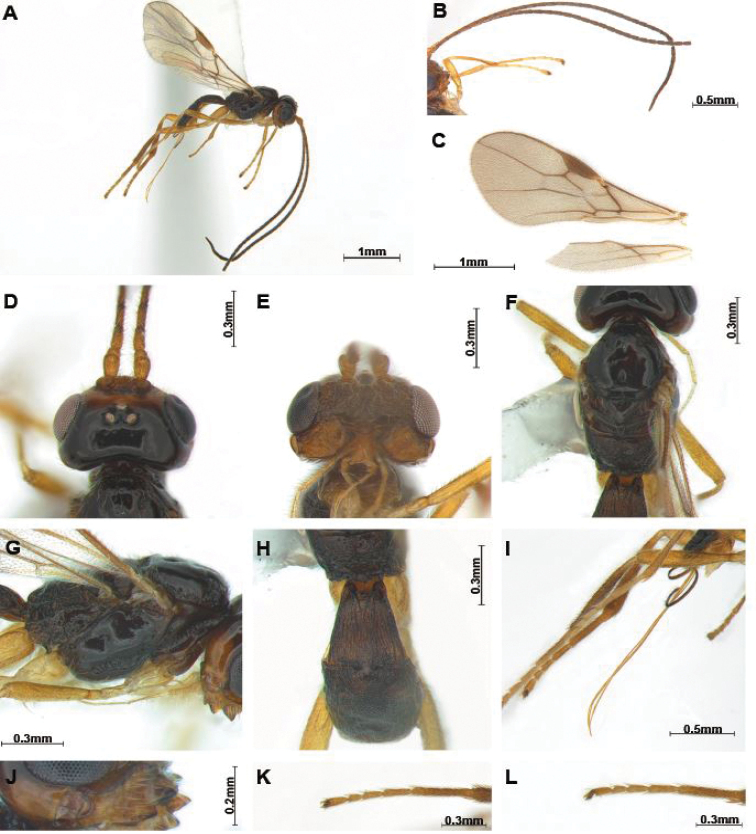
*Hylcalosiasutchanica* Belokobylskij, 1992 ♀ **A** body, lateral view **B** antennae **C** wings **D** head, dorsal view **E** head, ventral view **F** mesosoma, dorsal view **G** mesosoma, lateral view **H** propodeum and first metasomal tergite, dorsal view **I** ovipositor and hind leg, lateral view **J** mandible, lateral view **K** hind tarsus, dorsal view **L** hind tarsus, lateral view.

First flagellomere distinctly shorter than second (Figs 1B, 2B), eye slightly oval and glabrous, clypeus triangularly protruding anteriorly (Figs 1E, 2E), labrum small triangular shape, mandible with 3–4 teeth or lobes (Figs 1J, 2J), maxillary palp with 6 segments; notauli partially or completely present, scutellar sulcus distinct, precoxal sulcus complete (Figs 1G, 2G); fore wing (Figs 1C, 2C) vein 2-SR slightly bent, vein 2-SR shorter than vein 3-SR; hind wing vein 1-M longer than vein 1r-m; propodeum largely rugose (Figs 1F, 2F); second and third tergites rugose or granulated (Figs 1H, 2H); tarsal claws rather slender (Figs 1K,L; 2K,L).

#### Biology.

Unknown.

#### Distribution.

Palaearctic (East) and Oriental Regions.

### Key to species of *Hylcalosia* Fischer from Korea

**Table:** 

1.	Second metasomal tergite 1.4–1.5 times longer than third tergite (Fig. 3B); mesoscutum largely blackish or dark brown; medio-posterior depression of mesoscutum short (Fig. 2F); [vein r of fore wing comparatively long (Fig. 2C)]	***H.sutchanica* Belokobylskij, 1992**
–	Second metasomal tergite 1.1–1.2 times as long as third tergite (Fig. 3A); mesoscutum largely red or reddish-brown; medio-posterior depression of mesoscutum long (Fig. 1F)	**2**
2.	Head entirely black; vein r of fore wing comparatively short (Fig. 1C), 0.4 times as long as maximum width of pterostigma (Fig. 1C); metasoma largely reddish-brown; first metasomal tergite largely subparallel-sided (Fig. 1H); eye in dorsal view about 1.7 times longer than temple (Fig. 1D)	***H.bicolor* sp. nov.**
–	Head (except stemmaticum) brownish-yellow; vein r of fore wing medium-sized, 0.9 times as long as maximum width of pterostigma; metasoma black; first tergite gradually widened posteriorly; eye in dorsal view about as long as temple	***H.maetoi* van Achterberg, 1983**

### Hylcalosia bicolor

Taxon classificationAnimaliaHymenopteraBraconidae

Sohn & van Achterberg sp. nov.

159E8C8D-7F5B-5DB7-97B0-45549A806B1A

http://zoobank.org/CBFECB61-CB8E-4847-9F08-9B360554BEDE

Figures 1A–L

#### Type material.

***Holotype***. ♀ (KNA), South Korea, DMZ Botanical Garden, Mandae-ri, Haean-myeon, Yanggu-gun, Gangwon-do, 38°15'09.3"N, 128°06'40.6"E, 27 May–20 Jun 2017, Shin & Kim leg. GenBank accession no. MZ717196.

#### Comparative diagnosis.

This species is similar to *H.verticalis* Zhu, van Achterberg & Chen, 2018 from China because of the vertical vein r-m of fore wing, deep and coarsely crenulate notauli, eye much longer than temple in dorsal view and second tergite about as long as third tergite or slightly longer, but differs by having the hind tibia yellowish-brown (largely blackish in *H.verticalis*), the third metasomal tergite (except basally) largely smooth and, in lateral view, truncated apically (coarsely rugose and rounded apically in *H.verticalis*), vein 1-r-m of hind wing shorter than vein 1-M (about of equal length in *H.verticalis*), the pterostigma subparallel-sided apically (slightly widened in *H.verticalis*), vein 3-CU1 of fore wing comparatively short (long in *H.verticalis*) and the precoxal sulcus wide medially (comparatively narrow in *H.verticalis*).

#### Description.

♀. Length of body in lateral view 2.6 mm, length of antenna 4.7 mm and length of fore wing 2.9 mm.

***Colour*.** Body (Fig. 1A) mainly reddish-brown; head black; antenna brown basally; mandible reddish-brown. ***Head*.** Head (Fig. 1D) width 1.6 times median length in dorsal view. Antenna (Fig. 1B) 1.8 times longer than body in female, 43-segmented. First flagellomere 0.7 times longer than second. Eye slightly oval, 1.1 times as long as wide in lateral view. Width of face (Fig. 1E) 2.1 times its height from ventral rim of antennal sockets to upper margin of clypeus; face with long setae. Eye in dorsal view 1.7 times as long as temple. Ocello-ocular line (OOL) 4.5 times longer than diameter of anterior ocellus; OOL:antero-posterior ocellar line (AOL):postero-ocellar line (POL) = 18:6:7. Stemmaticum concave. Vertex smooth and shiny with groove. Mandible with four teeth or lobes (Fig. 1J); dorsal tooth large and lobe-shaped; ventral tooth lobe-shaped, middle of tooth curved. Medial length of mandible 1.7 times longer than maximum width. Labrum small, 1.4 times longer than wide. Maxillary palp 0.5 times longer than mesosoma.

***Mesosoma*.** Mesosoma (Fig. 1F) 2.0 times longer than wide in dorsal view: with medio-posterior depression; notauli coarsely crenulate anteriorly and deeply impressed, up to anterior level of medio-posterior depression (Fig. 1F); scutellar sulcus indistinct, with two carinae and sparse setae; small basal bump on hind coxa. Propodeum (Fig. 1F) largely reticulate, 1.7 times longer than wide in dorsal view. Metapleuron anteriorly crenulate and with setae; precoxal sulcus (Fig. 1G) crenulated, with about nine carinae. Fore wing (Fig. 1C) 2.4 times as long as wide; pterostigma long and robust, 3.4 times longer than wide; vein r of fore wing 2.6 times longer than wide and 0.4 times as long as maximum width of pterostigma; vein 1-M slightly bent; 2-SR+M not sclerotised; veins 1-SR+M:2-SR = 9:12; veins 2-SR: r: 3-SR = 12:3:7; first subdiscal cell of fore wing 2.8 times longer than wide. Hind wing veins M+CU:1-M:1r-m = 10:6:4.

***Leg.*** Hind coxa compressed, 1.5 times longer than hind trochanter; hind femur 0.7 times longer than hind tibia and 8.0 times longer than wide; hind tibia 1.1 times longer than hind tarsus, tarsal claws slender (Fig. 1L).

**Figure 3.**
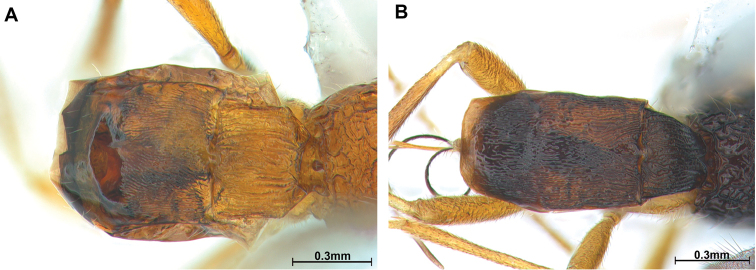
Metasoma, dorsal view **A***Hylcalosiabicolor* sp. nov. **B***Hylcalosiasutchanica* Belokobylskij, 1992

***Metasoma*.** First tergite parallel-sided posteriorly, striate and 1.1 times longer than its apical width (Fig. 3A); first tergite 0.9 times as long as second. Second tergite distinctly rugose and 1.2 times as long as third tergite, third tergite (except basally) largely smooth (Fig. 3A). Setose part of ovipositor sheath (Fig. 1I) 1.3 times longer than mesosoma, 1.3 times metasoma, 1.3 times as long as hind tibia, with long setae.

#### Male.

Unknown.

#### Distribution.

South Korea.

### Hylcalosia sutchanica

Taxon classificationAnimaliaHymenopteraBraconidae

Belokobylskij, 1992

E8858EF0-F396-538E-BE88-1C612BFF8810

Figures 2A–L

Hylcalosia sutchanica Belokobylskij, 1992: 148; 1998: 298; 2015: 538; Fischer, 2008: 722; Zheng et al., 2012: 455.
Hylcalosia adsimilis Papp, 1994: 139; Belokobylskij, 1998: 298; Fischer, 2008: 722; Yu et al., 2012; Zheng et al., 2012: 455. Synonymized by Belokobylskij (2015).

#### Material.

2♀ (KNA), South Korea, DMZ Botanical Garden, Mandae-ri, Haean-myeon, Yanggu-gun, Gangwon-do, 38°15'09.3"N, 128°06'40.6"E, 20 Jun–4 Jul 2017, Shin & Kim leg. GenBank accession no. MZ717194.

#### Re-description.

♀, length of body in lateral view 2.6–2.7 mm, length of antenna 4.1–4.3 mm and length of fore wing 2.7–2.9 mm.

***Colour*.** Body largely blackish; head entirely black dorsally and anteriorly brown, antenna reddish-brown and apically dark brown, mandible pale brown and apically dark brown; first tergite reddish-brown and mesonotum entirely blackish or dark brown.

***Head*.** Head (Fig. 2D) width 1.6 times median length in dorsal view. Antenna (Fig. 2B) 1.6 times longer than body, 40 or 42 segmented. First flagellomere 0.7 times as long as second, second flagellomere 1.1 times longer than third. Eye slightly oval, 1.1 times as long as wide in lateral view. Width of face (Fig. 2E) 2.0 times its height from ventral rim of antennal sockets to upper margin of clypeus; face with long setae. Eye in dorsal view 2.4 times as long as temple. Ocello-ocular line (OOL) 4.0 times longer than diameter of anterior ocellus; OOL:antero-posterior ocellar line (AOL):postero-ocellar line (POL) = 12:5:5. Vertex smooth and shiny with groove. Mandible (Fig. 2J) with four teeth and setae; dorsal tooth large and lobe-shaped and distinctly surpassing apex of first tooth, ventral tooth lobe-shaped, middle of tooth curved; second tooth narrow and sharp with dark brown tip and separated from first tooth by incision in lateral view. Medial length of mandible 1.7 times longer than maximum width. Labrum small, 1.3 times longer than wide. Maxillary palp 0.7 times as long as mesosoma.

***Mesosoma*.** Mesosoma (Fig. 2G) 2.1 times longer than wide in dorsal view; notauli moderately crenulated, but situated far from comparatively small medio-posterior depression (Fig. 2F); scutellar sulcus with four carinae; laterally mesopleuron and metapleuron with long setae, metapleuron distinctly rugose. Anterior half of propodeum smooth, posterior of median carina reticulate-rugose (Fig. 2H), lateral view of propodeum not curved dorsally; precoxal sulcus (Fig. 2F) shallow and with 14 crenulae. Fore wing (Fig. 2C) 2.4 times longer than wide; pterostigma long and thick, 3.4 times longer than wide; vein r of fore wing 2.9 times longer than wide; vein 2-SR slightly bent; vein 2-SR+M and r-m not sclerotised; veins 2-SR: r: 3-SR = 11:2:7; first subdiscal cell of fore wing 1.5 times longer than wide. Hind wing veins M+CU:1-M:1r-m = 11:7:5.

***Leg.*** Hind coxa smooth and 1.2 times longer than hind trochanter; hind femur 0.9 times as long as hind tibia and 8.5 times longer than wide; hind tibia 0.9 times longer than hind tarsus, tarsal claws slender (Fig. 2L).

***Metasoma*.** First tergite gradually widened posteriorly (Fig. 3B), striate and comparatively narrow, 1.1 times longer than its apical width; first tergite 0.8 times as long as second tergite, second tergite 1.4–1.5 times longer than third and largely rugose and third tergite largely rugose (Fig. 3B). Setose part of ovipositor sheath (Fig. 2I) 1.5 times longer than mesosoma, 1.7 times longer than hind tibia and with medium-sized setae.

#### Distribution.

Russia, China, Korea.

## Supplementary Material

XML Treatment for Hylcalosia

XML Treatment for Hylcalosia bicolor

XML Treatment for Hylcalosia sutchanica
